# Direct pulmonary delivery route in lung cancer: a highway for siRNA therapeutics

**DOI:** 10.3389/fonc.2025.1722906

**Published:** 2026-01-23

**Authors:** Muhammad Shakeeb Sharif, Luca Luzzi, Michelino De Laurentiis, Antonio Giordano, Marcella Barbarino

**Affiliations:** 1 Department of Clinical and Translational Oncology, Scuola Superiore Meridionale, Naples, Italy; 2 Department of Medical Biotechnology, University of Siena, Siena, Italy; 3 Thoracic Surgery Unit and Lung Transplant, Siena University Hospital, Siena, Italy; 4 Department of Breast and Thoracic Oncology, Istituto Nazionale Tumori Istituti di Ricovero e Cura a Carattere Scientifico (IRCCS) “Fondazione G. Pascale”, Naples, Italy; 5 Sbarro Institute for Cancer Research and Molecular Medicine, Center for Biotechnology, College of Science and Technology, Temple University, Philadelphia, PA, United States

**Keywords:** bronchoscopy, direct drug delivery, EBUS-TBNI, inhalation, lung cancer, RNA silencing

## Abstract

Direct drug delivery encompasses minimally invasive methods for the local administration of therapeutics and is already widely applied in diseases affecting the liver, eyes, peritoneum, breast, joints, coronary arteries, and brain. In oncology, localized approaches such as intratumoral injections, implantable depots, inhalable aerosols, and image-guided procedures allow controlled drug release at tumor sites with high selectivity. Given its large surface area, rich vascularization, and anatomical accessibility, the respiratory system provides an ideal setting for direct delivery strategies across a range of respiratory diseases. Among these, lung cancer, the leading cause of cancer-related deaths worldwide, is characterized by high molecular heterogeneity, making it particularly suitable for targeted gene-silencing and replacement therapies at specific oncogenic drivers. Advances in genomics and transcriptomics increasingly support the potential of gene therapy, especially RNA-based therapeutics and gene-editing technologies, to selectively silence or correct oncogenic mutations. In patients with unresectable or recurrent disease, where therapeutic precision is crucial, the combination of direct pulmonary drug delivery and RNA-based therapies offers a powerful synergy: anatomical precision, reduced systemic toxicity, and molecular selectivity targeting tumor-specific alterations. Unlike previous reviews, this work provides an integrated perspective that bridges findings from orthotopic *in vivo* studies on siRNA nanomedicine with emerging clinical evidence, underscoring direct pulmonary delivery as a central strategy in precision medicine for lung cancer. By critically examining advanced delivery technologies, the review considers both their potential advantages and the scientific and technical challenges that remain in the clinical translation of siRNA for lung cancer. Moreover, by highlighting how direct pulmonary administration can overcome these challenges, it underscores the transformative potential of siRNA therapeutics in lung cancer and the need for sustained, high-intensity collaboration between scientists and clinicians to advance RNA-based therapies in thoracic oncology.

## Graphical Abstract

**Figure:**
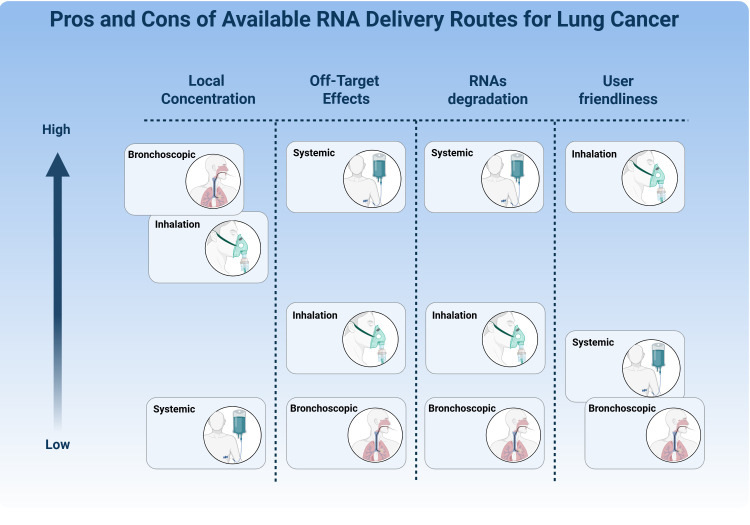
Pro and Cons of available RNA delivery routes for lung cancer.

## 1 Introduction

Direct drug delivery (DDD) refers to minimally invasive methods for the local administration of therapeutics, actually widely adopted for the treatment of many diseases including those affecting the liver, eyes, peritoneum, breast, joints, coronary arteries, and brain. In oncology, techniques such as intratumoral injections, implantable depots, inhalable aerosols, and image-guided procedures are used to achieve localized, controlled-released exposure to drugs, with reduced side-effects related to whole body exposure (1–4). The anatomical and physiological characteristics of the respiratory system provide a strategic advantage for localized treatments. The large surface area of the alveolar epithelium, coupled with its rich vascularization and the direct accessibility of the upper airways facilitate efficient deposition and absorption of therapeutic agents. In the context of lung cancer (LC), local delivery is currently implemented through interventional bronchoscopy and, in selected patients with advanced diseases, *via* intraoperative administration of high-dose chemotherapy into the thoracic cavity (Hyperthermic Intrathoracic chemotherapy; HITHOC) (5, 6). Although percutaneous intratumoral drug delivery has also been explored, particularly in patients with peripheral lesions or poor pulmonary function, its use remains limited for its invasive nature and for the associated risks such as pneumothorax and bleeding. Given these limitations, bronchoscopic and inhalation-based approaches are increasingly favored as direct drug delivery routes for their minimally invasive nature.

LC is classified in two principal histological subtypes, small-cell lung cancer (SCLC) and non-small cell lung cancer (NSCLC), characterized by specific molecular alterations that influence disease progression, treatment response, and prognosis (7, 8). Driver mutations in KRAS, BRAF, MET, and HER2, activating alterations in EGFR, and rearrangements in ALK and ROS1 genes, define distinct molecular subtypes with specific therapeutic susceptibilities (9). Additionally, loss-of-function mutations in TP53 are frequently observed contributing to genomic instability and resistance to conventional therapies (9). This molecular heterogeneity has catalyzed the development of targeted therapies, such as tyrosine kinase inhibitors (TKIs), which have significantly improved clinical outcomes in selected subsets of patients (10–12). Insights into the genomic and transcriptomic landscape of NSCLC are even more supporting the value of gene therapy approaches, including RNA-based therapeutics and gene-editing technologies, designed to silence or correct pathogenic mutations.

Despite its promise, the clinical translation of gene therapy progressed slowly because of challenges related to nucleic acids stability, retention, and delivery vector safety. Viral vectors, historically the predominant delivery platforms, leverage specific capsid or envelope proteins to overcome cellular barriers and achieve high transduction efficiencies. Nonetheless, their use is constrained by challenges such as uncontrolled biodistribution, immunogenicity, and the inherent risk of insertional mutagenesis arising from genomic integration. Recently, innovations in biomaterials and nano- and micro-technologies, have led to the development of new non-viral delivery systems able to answer these limitations (13). Lipid nanoparticles (LNPs) (14), N-acetylgalactosamine (GalNAc) conjugates (15), microrobotic swarms (16), biodegradable polymers (17), and stimuli-responsive nanoparticles (NP) (18), are renewing the interest in gene- and RNA -based therapies including mRNAs, small interfering RNA (siRNAs), microRNAs (miRNA) and antisense oligonucleotides (ASO). Compared with viral vectors, NP formulations provide a safer, cost-efficient, and scalable platform for nucleic acid delivery, without requiring specialized biosafety facilities (19).

SiRNA technology has rapidly emerged as a leading strategy among other RNA-based approaches, owing to its superior stability, high target specificity, and relatively simple and cost-effective synthesis (20–23). The localized administration of siRNA may overcome key challenges linked to systemic delivery, and improve RNA uptake at the cellular level, especially when supported by advanced carrier systems (24). For LC, DPDD of siRNA represents an opportunity to realize the long-awaited clinical translation of RNA-based therapies for the targeting of driver and acquired mutations.

Inhalation as a delivery route has been used for over two thousand years, with its origins tracing back to ancient civilizations that recognized the therapeutic value of inhaled substances (25). Over the centuries, this route of administration has evolved substantially and is now a cornerstone in the management of respiratory diseases. Its clinical and commercial relevance continues to expand, with current projections estimating that the global market for inhalable drugs will grow at a Compound Annual Growth Rate (CAGR) of 6.31% during the forecast period from 2024 to 2030 (26), a growth driven by technological innovations, broader therapeutic applications, and the increasing demand for non-invasive, targeted delivery systems (27, 28). Despite their user-friendly characteristics, inhaled nanoparticle-based therapeutics still present several limitations. A major constraint is the particle-size–dependent deposition pattern within the respiratory tract: larger particles (~10 μm) primarily deposit in the upper airways, medium-sized particles (5–10 μm) tend to accumulate in the central lung regions, whereas smaller particles (1–5 μm) can reach the lower airways more easily but may also be exhaled before deposition (29). The pulmonary deposition of inhaled nanoparticles is further affected by their electrostatic charge, typically quantified as the zeta potential, which affects their aerodynamic behavior and effective particle size. Electrostatic interactions regulate both dispersion and agglomeration and therefore must be carefully controlled to ensure therapeutic efficacy. Furthermore, inhaled particles are cleared from the lungs by excess mucus production and phagocyte-mediated mechanisms. Other limitations are those related to undesired effects such as irritation of upper airway tissues, enzymatic degradation of biomolecules by airways defense, immunogenicity and differences in breathing patterns among patients (28). To address these limitations, nanocarriers are being engineered through size optimization, surface and compositional modifications, and the selection of excipient formulations that limit aggregate formation and shield nanoparticles from enzymatic degradation, often in combination with mucolytic agents to enhance mucus penetration (30, 31). In addition, nanoparticles have been functionalized with bispecific antibodies (bsAbs), enabling dual-pathway targeting, a complementary therapeutic strategy that can improve cellular internalization, enhance site-specific delivery, and yield meaningful clinical benefits (32–34).

**Figure 1:**
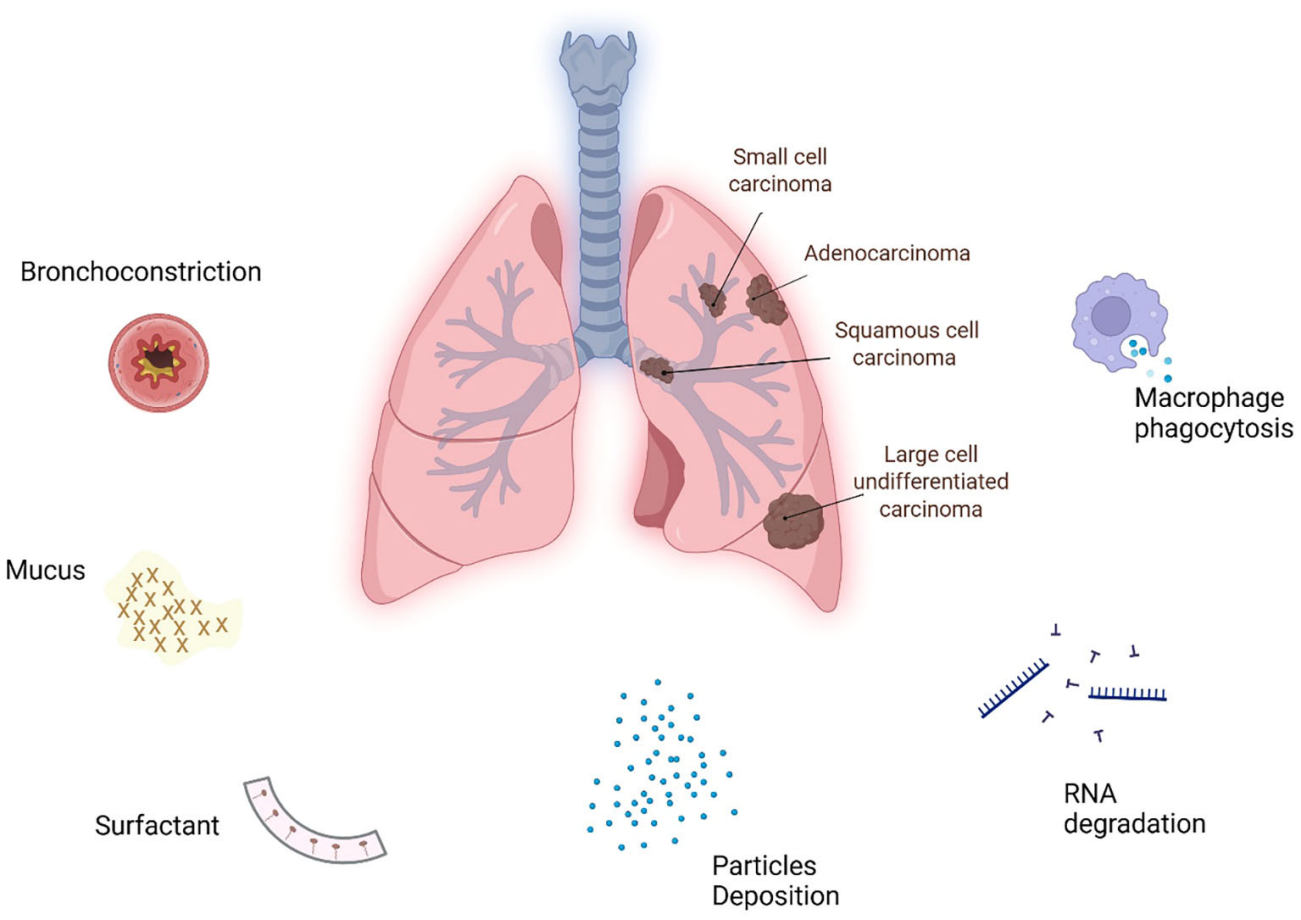
Major barriers to RNA-loaded nanoparticles delivery to lung.

Though less widely adopted, bronchoscopic delivery represents an alternative strategy for localized drug delivery, with a strong therapeutic potential in LC. It has historical roots dating back to 1922, when Yankauer used a rigid bronchoscope to place radium capsules into a bronchogenic carcinoma, laying the foundation for therapeutic bronchoscopy (35). Unlike inhalation, endobronchial drug delivery allows direct access to distal disease sites within the lungs, bypassing several limitations associated with inhalation-based therapies, including particles deposition in the upper respiratory tract, mucociliary clearance, irritation of upper airway tissues, and enzymatic degradation of biomolecules (28, 36, 37). Importantly, it overcomes the challenge of unpredictable drug concentrations resulting from interindividual differences in ventilatory patterns, which can significantly affect aerosol distribution and therapeutic efficacy (38). Recent technological refinements of the bronchoscope have further expanded its scope in the clinical management of LC, positioning it as a valuable addition to routine clinical protocols. Today, interventional bronchoscopy is an important part of the local treatment of lung cancers (5). Major barriers to RNA-loaded NPs delivery to the lung are summarized in **Figure 1** (Created in BioRender. Arshid, N (2025). https://BioRender.com/97wwwcu).

Despite the consolidated clinical use of inhalation therapy and therapeutic bronchoscopy, and the demonstrated efficacy of nucleic acid-based treatments across several diseases, with more than 150 RNA-LNPs in development to date (14), no clinical trials in the past five years have evaluated DPDD platforms for siRNA delivery in LC. This highlights a critical gap between technological progress and its clinical translation in oncology, an unmet need that demands focused attention. While previous works have extensively examined inhalation therapy, therapeutic bronchoscopy, advanced drug delivery carriers, and RNA-based therapeutics as distinct areas of investigation, a comprehensive analysis integrating these domains within the framework of DPDD of siRNA for LC is currently lacking. This study aims to fill that gap by reviewing evidence from the past five years on the therapeutic potential of integrating DPDD and RNA-based strategies for lung cancer, identifying the scientific and technological gaps that have so far hindered the clinical translation of siRNA, and outlining opportunities to facilitate this transition and broaden the therapeutic landscape in pulmonary oncology.

The first part of the review presents the results from studies investigating the efficacy of drug delivery through direct pulmonary routes focusing exclusively on orthotopic *in-vivo* models of LC, as they represent the most relevant preclinical setting for this purpose. We then examine recent investigational studies exploring local drug administration in lung cancer *via* both inhalation and bronchoscopic approaches. Finally, siRNA candidates that have entered clinical trials for oncological diseases are reviewed.

Driven by accelerating progress in DPDD and siRNA therapeutics within oncology, this review focuses on studies published over the past five years to ensure alignment with current translational advances. Collectively, the emerging data indicate that both approaches, individually and in combination, hold significant potential to improve therapeutic precision in lung cancer.

This review aims not only to map the current state of the art, but also to outline strategies that may accelerate the convergence of these two promising domains into next-generation respiratory oncology.

## 2 Direct pulmonary drug delivery in orthotopic lung cancer models

*In-vivo* models represent an indispensable step for clinical translation, as regulatory approval for initiating any clinical trial requires preclinical evidence of efficacy, safety, biodistribution, and tolerability. Among the available *in-vivo* approaches, orthotopic models constitute the most appropriate platform for evaluating DPDD strategies, as they preserve the native anatomical architecture and the microenvironment of the lungs (39). Unlike ectopic or subcutaneous systems, orthotopic models allow for a more accurate assessment of intrapulmonary drug dispersion, retention, and bioavailability within the diseased organ, also preserving the local tissue remodeling, interactions with the pulmonary vasculature, and the crosstalk with immune and stromal components when immunocompetent animals are used.

Murine models are the most widely used in preclinical research due to their low cost, ease of handling, and compatibility with high-throughput studies. In the context of lung cancer, orthotopic rodents models have been progressively refined to investigate DPDD strategies (39), particularly *via* inhalation and intratracheal instillation, while bronchoscopic techniques have been explored in a limited number of preclinical studies, primarily involving rats, mice and pigs, for the evaluation of drug concentration, or for tumor visualization, rather than for the assessment of its therapeutic potential.

The following section reviews recent findings on the direct delivery of cancer therapeutics in orthotopic models of lung cancer *via* inhalation and bronchoscopy, providing evidence of the translational potential of DPDD strategies for LC treatment.

### 2.1 Direct pulmonary drug delivery through inhalation

Chemotherapeutics, biologics and nanocarriers have been investigated as inhalable formulations in recent years.

Classical chemotherapeutic drugs such as cisplatin and topotecan have shown promising results when administered *via* inhalation. Dry powder cisplatin (CIS-DPI) direct delivery has demonstrated to increase Programmed Death-Ligand 1 (PD-L1) expression in mice, and to induce a synergic response in tumor regression with anti-PD-1 immunotherapy (40). Similarly, inhaled topotecan at 1 mg/kg demonstrated superior antitumor efficacy in orthotopic rat model compared to a 5 mg/kg intravenous (IV) administration, reducing tumor burden by 88% (p < 0.00001). The longer systemic half-life of inhaled topotecan at lower dose, compared to higher dose IV administration, was further demonstrated in subcutaneous xenograft models bearing A549, H358, and H1975-derived tumors, where was observed a reduction in tumor weight ranging from 4.6% to 8.8% (41).

Similarly, the direct delivery of biologics combined with immunotherapeutics has demonstrated synergic effects. Aerosolized administration of hesperidin-loaded nanoparticles (HNPs) in combination with anti-CD40 immunotherapy elicited selective tumor uptake and improved survival in mice (42). Additionally, inhalable exosome-based platforms derived from CAR-T cells (CAR-Exos) engineered to express anti-mesothelin, have demonstrated immunomodulatory efficacy and good tolerability in mesothelin-positive lung cancer, further supporting the potential of complex biologics for delivery through the inhalation route (43).

The use of nanocarriers to exploit tumor-specific vulnerabilities is emerging as a rapidly expanding field. Macrophage membrane-coated nanoreactors (DHA-N@M), administered *via* nebulization in mice achieved tumor deposition levels approximately 70-fold higher than IV delivery. When combined with X-ray irradiation, these nanoparticles released nitric oxide (NO), which synergized with radiation-induced reactive oxygen species (ROS) to generate peroxynitrite (ONOO^-^), triggering ferroptosis and resulting in 93% tumor inhibition (44). Additionally, inhalable nanotherapies have been investigated for targeting cancer stem cells (CSCs) through immunometabolic modulation. Iron-based nanoparticles functionalized with dextran have been shown to accumulate into pulmonary microlesions of mice disrupting metabolic and redox homeostasis, upregulating CD44, and inducing ferroptotic cell death (45). Furthermore, up-conversion nanoparticle (UCNP)-based nanocages, functionalized with VEGF-siRNA and AS1411 aptamer, showed promising results in orthotopic LC mouse models, with reduced tumor proliferation, improved survival, and stable systemic tolerability (46).

### 2.2 Direct pulmonary drug delivery through bronchoscope

Few studies have investigated the feasibility and efficacy of DPDD through bronchoscope in orthotopic LC models. The reason mainly lies in the technical limitations related to the small size of murine airways (47). In the last five years, only one study investigated bronchoscope delivery in mice, demonstrating the ability of this technique in obtaining uniform drug distribution (48).

To overcome small rodents’ anatomical limitations, porcine cancer models have been developed for their close resemblance to human pulmonary architecture, including similar airway branching patterns, lobular organization, and immune responses. Engineered “Oncopigs” genetically modified to express human-relevant mutations such as KRAS-G12D and TP53-R167H, have been developed as a model for replicating key features of the tumor microenvironment, thus representing a living model e for the validation of *in-vitro* results, in particular for viral vectors and CRISPR/Cas9-based gene editing studies (49).

In parallel, bronchoscopic alpha radiotherapy has been investigated as a localized intervention *via* implantation of micro sources of Radium-224 (Alpha Diffusing Alpha-emitters Radiation Therapy) in porcine lungs. The study demonstrated precise deposition in clusters ≤4 mm apart, with no systemic toxicity, no device migration, and minimal local fibrotic or inflammatory reactions (50).

## 3 Clinical investigations of direct pulmonary drug delivery for lung cancer

To date, two minimally invasive pulmonary delivery routes have demonstrated clinical applicability in humans: inhalation and endobronchial administration.

Inhalation route, initially developed for chronic respiratory diseases such as asthma, chronic obstructive pulmonary disease (COPD) and pulmonary fibrosis (51, 52), is now widely employed for the administration of different classes of compounds such as bronchodilators β_2_-agonists (e.g., salbutamol, formoterol), corticosteroids (e.g., budesonide), antibiotics (e.g. tobramycin and colistin), to manage different pulmonary conditions (51–54). More recently, the inhalation has gained increasing interest for the delivery of biologics, including interferons and monoclonal antibodies, in the context of viral respiratory infections such as SARS-CoV-2 (55).

In contrast, bronchoscopic drug delivery remains at the investigational stage. Although less extensively studied than inhalation, bronchoscopic administration is emerging as a promising alternative for targeted therapy in lung cancer.

In the following section, we review recent clinical studies evaluating chemotherapeutic delivery *via* inhalation and bronchoscopy for the treatment of lung cancer.

### 3.1 Direct pulmonary drug delivery through inhalation

Inhalation as a delivery route for LC management has demonstrated many advantages compared to systemic administration, including improved drug retention, low systemic toxicity, and user-friendliness (56). Approved inhaled drugs include aerosolized and dry powder formulations (57) delivered through devices such as nebulizers, dry powder inhalers (DPIs), pressurized metered-dose inhalers (pMDIs), and pressurized breath-activated inhalers (BAIs) able to synchronize dose delivery with the breathing process (58).

Although inhaled chemotherapy is not devoid of risk, adverse events reported in the literature are infrequent and predominantly attributable to administration-related factors (54). Preclinical studies have demonstrated encouraging results with inhaled formulations of chemotherapeutics, biologics, and RNA-based agents (59–61) but, over the past five years, only five new clinical trials have been started to study the inhalation route for cancer therapy (**Table 1**), reflecting the delayed progression from preclinical models to clinical trials.

**Table 1:** Investigational inhaled therapy (2020–2025).

Drug	Primary indication	Administration route	Clinical phase	Trial ID (NCT)	Outcome/recruitment status	Year
Cisplatin	Stage IV NSCLC	Inhalation	Phase I/II	NCT06896890	MTD; Recruiting	2023
Azacytidine + SOC + Durvalumab	Resectable NSCLC	Inhalation	Phase I/II	NCT06694454	Phase I: MTD; Not yet Recruiting Phase II: pCR Not yet Recruiting	2024
MD006 (mRNA vaccine) + PD-1 therapy	LC	Inhalation	Phase I	NCT06928922	MTD; Recruiting	2025
Aztreonam and Vancomycin + SOC	NSCLC	Inhalation	Phase I	NCT05777603	DLT; Recruiting	2023
Azacytidine + Bintrafusp Alfa IV	Pulmonary metastases	Aerosol	Phase I/II	NCT04648826	MTD, DLT, PK, ORR. Withdrawn	2021

*SOC, standard of care; NSCLC, non-small cell lung cancer; LC, lung cancer; PD-1, Programmed Cell Death 1 Protein; MTD, Maximum Tolerated Dose; pCR, pathologic complete responses; DLT, Dose Limiting Toxicity; PK, Pharmacokinetics; ORR, Objective Response Rate.

### 3.2 Direct pulmonary drug delivery through bronchoscope

Originally developed as a diagnostic tool for visualizing and sampling the airways, bronchoscopy has progressively adopted for therapeutic interventions including airway stenting and transbronchial biopsy (62), and for the localized delivery of drugs in infectious diseases, inflammatory conditions, and airway obstruction (63, 64). In oncology, interventional bronchoscopy has expanded dramatically, with clinically validated applications including photodynamic therapy and endobronchial brachytherapy (5).

Bronchoscopy precision was further improved in 2001 by Herth and colleagues with the introduction of the convex probe endobronchial ultrasound (EBUS) for mediastinal lymph node sampling (65). The integration of dedicated aspiration needles with real-time ultrasound guidance has enabled the safe and accurate sampling of lymph nodes and lesions smaller than 1 cm, which were previously inaccessible using conventional bronchoscopic techniques (66).

Another milestone came in 2015, with the introduction of endobronchial ultrasound–guided transbronchial needle injection (EBUS-TBNI) for the localized administration of chemotherapy in recurrent NSCLC, opening new perspectives for targeted drug delivery to distant lung cancer lesions (67–69). This technique has demonstrated a low incidence of complications, an aspect particularly critical for patients with impaired pulmonary function who are unable to tolerate standard therapeutic options such as surgery, systemic chemotherapy, or radiotherapy (70). EBUS-TBNI has demonstrated unique advantages in the treatment of malignant lymph nodes and parenchymal lesions, enabling highly precise drug delivery, reduced therapeutic dosing, and significant modulation of the tumor immune microenvironment (71, 72). It also allows for multi-site injections within a single tumor, enhancing intratumoral drug dispersion. Moreover, computational models have been validated to estimate the appropriate dose as a function of tumor volume (69, 73). Consequently, EBUS is gaining recognition as a valuable, minimally invasive platform for targeted therapy.

Technological advances have further improved bronchoscopic drug delivery. Electromagnetic navigation bronchoscopy (ENB) integrates CT-derived virtual airway mapping with real-time electromagnetic tracking, enabling access to small, difficult-to-reach pulmonary nodules (74). Building upon the principles of ENB, robotic-assisted bronchoscopy (RAB) has enhanced stability and precision through the use of a robotic articulated bronchoscope associated with a 3D image reconstruction for real-time endobronchial navigation. Systems such as Monarch™, Galaxy™ and Ion™, with a superior localization accuracy, allow operators to reach narrower airways and perform complex procedures with greater control (75). Robotic ultrathin bronchoscopy, which employs optical fibers with an outer diameter ≤ 3.0 mm, can even reach distal airways up to the 12th bronchial generation, with great potential to become a key enabler of precision medicine in peripheral lesions (76).

Since 2020, six phase I clinical trials have been initiated to investigate local drug administration *via* EBUS-TBNI in lung cancer (**Table 2**). Although limited in number, these studies reflect growing interest in this approach as a valuable alternative to conventional routes of administration. Among these, three studies (NCT04311762, NCT04809103, and Khan et al., 2024 (77)) specifically focus on intratumoral cisplatin delivery, providing evidence of feasibility and safety in both early and advanced-stage disease (78). Preliminary data from the study of Khan and coll. demonstrated that a single 20 mg intratumoral dose of cisplatin was well tolerated in patients with stage IV NSCLC, with no dose-limiting toxicities and plasma platinum levels approximately 100-fold lower than those observed with IV administration. Although the trial was halted early due to emerging immunomodulatory data, it confirmed the procedural feasibility of a “diagnose-and-treat” paradigm.

**Table 2:** Investigational bronchoscopic intratumoral chemotherapy (2020–2025).

Drug	Primary indication	Administration route	Clinical phase	Trial ID	Outcome/recruitment status	Year
Cisplatin	Resectable NSCLC	Bronchoscopy with cone-beam CT confirmation; IT	Phase I	NCT04809103	DLT; Recruiting	2021
Cisplatin	Stage IV LC	EBUS-TBNI; IT	Phase I	NCT04311762	DLT: Active, not recruiting	2020

NSCLC, Non-Small Cell Lung Cancer; LC, lung cancer; CT, Computed Tomography; IT, intratumoral; EBUS-TBNI, Endobronchial Ultrasound-Transbronchial Needle Injection; DLT, Dose Limiting Toxicity.

Additionally, nanoparticulate paclitaxel (NanoPac; NCT04314895) is under investigation in non-resectable LC (advanced stages II, III and IV), and anti-PD-L1 nanobody Envafolimab in metastatic/recurrent NSCLC in combination with chemotherapy (NCT06108726).

Remarkably, in April 2025 the world’s first gene therapy study started for the local delivery of Adeno-Associated Virus in KRAS mutant NSCLC *via* bronchoscopy or CT-guided percutaneous injection (NCT06934590**).**

## 4 siRNA therapeutics for lung cancer

To date, six siRNA-based drugs have received regulatory approval, all targeting non-oncological indications. According to recent data (23), over 380 clinical trials are currently underway investigating siRNA therapies for non-oncology conditions, with a strong focus on metabolic and genetic disorders.

In contrast, the application of siRNA therapeutics in oncology remains relatively limited. Between 2004 and 2024, approximately 40 clinical trials have been estimated to have been initiated (23), most of which focus on solid tumors and are mainly in phase I of development, with only a few advancing to phase II evaluation. However, the study provides limited detail on these oncology-specific trials, making it challenging to ascertain how many specifically address lung cancer. Another study identified four clinical trials launched before 2020 targeting lung cancer, for which results are still pending (79).

Although five siRNA-based candidates have entered clinical evaluation in the past five years, only four are distinct entities. Two of them, STP705 and STP707, share the same molecular composition but differ in their delivery routes, being formulated respectively for intratumoral and intravenous administration for various solid tumors. STP705 is currently involved in four phase I–IIb trials (NCT04844983, NCT05421013, NCT04669808, NCT04676633), while STP707 is being tested in two phase I studies (NCT05037149, NCT06424301). Both formulations utilize a proprietary Histidine-Lysine Polymer (HKP) nanoparticle system for non-viral delivery, targeting TGF-β1 and COX-2. Phase I trial NCT05037149 reported a favorable safety profile and signs of disease stabilization in a substantial proportion of evaluable patients (80). STP705 is also under evaluation in non-oncological settings including keloid recurrence prevention post-excision (NCT04844840; Phase II), for wound healing and fibrosis modulation (NCT04844879; phase I), for hypertrophic scars in a dose-escalation phase I/II study (NCT05196373) and in regenerative and aesthetic medicine with promising results in a Phase I trial (NCT05422378) (81). Other siRNAs are being investigated in diverse therapeutic areas. In retinoblastoma, an intraocular siRNA targeting nudt21 is currently being evaluated in a phase I trial (NCT06424301). For polycythemia vera, SLN124, a double-stranded siRNA designed to silence TMPRSS6 mRNA, is under investigation in a phase I/II study (NCT05499013). Additionally, in the context of relapsed or refractory B-cell non-Hodgkin lymphoma, the investigational molecule CpG-STAT3 siRNA CAS3/SS3, which combines a CpG oligonucleotide with a siRNA in a single construct, is being tested *via* intratumoral injection in combination with localized radiation therapy in a phase I trial (NCT04995536). Investigational siRNA studies in oncology are summarized in **Table 3**.

**Table 3:** Investigational RNA-based therapies in oncology (2020–2025).

Drug	RNA type/technology	siRNA target(s)	Primary indication	Delivery route	Clinical phase	Trial ID (NCT)	Outcome/recruiting status	Year
STP705	siRNA via HKP nanoparticles	TGF-β1, COX-2	Cutaneous isSCC, HCC, BCC	ILI	Phase I/II/IIb	NCT04844983 NCT04669808 NCT04676633 NCT05421013	LTD AEs SAEs EOT Completed/no results posted	2021 2020 2020 2022
STP707	siRNA via HKP nanoparticles	TGF-β1, COX-2	ADT	IVI	Phase I	NCT05037149 NCT06424301	TEAEs DLT Active, not recruiting	2021 2021
SLN124	GalNAc-conjugated siRNA	TMPRSS6	PV	SCI	Phase I/II	NCT05499013	AEs SAEs Recruiting	2022
CpG-STAT3 siRNA CAS3/SS3	siRNA CAS3/​SS3	STAT3	B-cell NHL	ILI	Phase I	NCT04995536	IAEs DLT Withdrawn	2024
NUTD21 siRNA	siRNA	NUDT21	RB	IO	Phase I	NCT06424301	TEAEs Recruiting	2024

siRNA, small interfering RNA; HKP, histidine–lysine polymer; TGF-β1, transforming growth factor beta 1; COX-2, cyclooxygenase-2; TMPRSS6, Transmembrane protease, serine 6; STAT3, signal transducer and activator of transcription 3; NUDT21, Nudix Hydrolase 21; isSCC, *in situ* squamous cell carcinoma; BCC, Basal Cell Carcinoma; HCC, Hepatocellular Carcinoma; NUDT21, Nudix Hydrolase 21; ADT, Advanced solid tumors; PV, Polycythemia vera; RB, Retinoblastoma; B-cell NHL, B-cell Non-Hodgkin Lymphoma; ILI, Intralesional Injection; IVI, Intravenous Infusion; SCI, Subcutaneous Injection; IO, Intraocular; EOT, End of Treatment; SAEs, Serious Adverse Events; IAEs, Incidence of Adverse Events; DLT, Dose Limiting Toxicity; TEAEs, Treatment-Emergent Adverse Events.

## 5 Discussion

LC remains a leading cause of cancer-related mortality worldwide (82). In patients with unresectable or recurrent disease, therapeutic precision is critical. In this context, direct local delivery and RNA-based gene therapies hold promises as transformative approaches to improve both survival and quality of life. On the one hand, DPDD enables anatomical precision lowering systemic toxicity; on the other, RNA therapies allow selective targeting of molecular dysregulations specific to tumor cells, thereby sparing healthy tissues. These features are particularly relevant for peripheral and deep-seated NSCLC lesions characterized by targetable genetic alterations, allowing high specificity for the patient’s molecular tumor profile.

Despite the availability of well-established pulmonary delivery routes, the proven efficacy of RNA-based therapies across multiple diseases, and continuous innovation in nucleic acid delivery systems, several translational barriers persist. By reviewing advances from the past five years, addresses the therapeutic potential of direct pulmonary delivery (DPDD) platforms for siRNA in lung cancer, integrating evidence from preclinical *in vivo* studies and reporting recent clinical investigations across different siRNA administration routes.Through this approach, the review guides the reader through a complex and multidisciplinary field, identifying the scientific and technological gaps that have so far hindered the clinical translation of siRNA, and outlining opportunities to facilitate this transition and broaden the therapeutic landscape in pulmonary oncology.

1. Preclinical Models

A recurring limitation emerging from our analysis lies in the scarcity of preclinical *in-vivo* models that can realistically recapitulate the human respiratory system. While their absence has not halted progress, more physiologically relevant models could markedly accelerate clinical translation of DPDD research. Orthotopic murine models have been progressively refined for DPDD research (39). However, commonly used methods such as intratracheal instillation, while suitable for experimental studies, do not accurately replicate clinical practice, and bronchoscopic approaches remain technically challenging to implement in small animals. Large animal models, such as the recently developed Oncopigs (49), offer closer anatomical and physiological similarity to humans and may represent a valuable option, but ethical and economic constraints limit their use. For the growing recognition that animal welfare must be treated as a priority in preclinical research and in line with the 3Rs principle (Replacement, Reduction, Refinement) (83–85), recent years have seen the development of an expanding range of experimental strategies that offer physiologically relevant and potentially more predictive alternatives to animal models.

Organ- and tumor-on-a-chip technologies, including 3D-bioprinted microenvironments and co-cultures incorporating immune, endothelial, and stromal cells have been progressively refined precise control of biomechanical cues, blood-flow-like perfusion, and drug exposure (84). Notably, the lung-on-a-chip system faithfully recreates the human alveolar–capillary interface, including the air–liquid interface and breathing-like mechanical strain (86).

In parallel, patient-derived *ex-vivo* lung tumor slices have been developed to investigate microenvironmental responses to immunotherapy. By introducing tumor and immune cells into healthy lung slices, these models effectively reproduce the complexity and heterogeneity of the lung tumor microenvironment (TME), providing a physiologically relevant and versatile platform for lung cancer research and therapeutic discovery (87).

Artificial intelligence (AI) is also transforming drug development by leveraging large-scale human datasets to generate more accurate and human-specific predictions. AI models can screen thousands of compounds in parallel and deliver results more rapidly than traditional, resource-intensive approaches (88). Moreover, AI-based models have demonstrated accuracy in predicting nanoparticle transport, immune interactions, barrier responses, and in contributing to the rational design of intelligent nanocarriers positioning them as powerful, low-cost tools capable of replacing or substantially reducing animal use in pulmonary research and disease modeling (89).

2. Pulmonary delivery

Although intratracheal and intranasal administration have been extensively studied in preclinical models for siRNA delivery in inflammatory and respiratory diseases, their clinical translation to thoracic oncology remains unexplored (90, 91).

Inhalation is the most widely adopted clinically established pulmonary delivery route, supported by well-defined regulatory frameworks and technologies approved for non-oncological indications such as asthma, COPD, and pulmonary infections (51, 52). Although inhalation is an extensively investigated delivery route, with numerous preclinical and early clinical studies, its use in lung cancer therapy remains limited mainly due to safety concerns related to the intrinsic toxicity of chemotherapeutics, which require more stringent evaluation than non-mutagenic inhaled drugs. Patient-dependent factors, including airway geometry, respiratory dynamics, and mucociliary clearance, introduce additional complexity by making drug concentrations difficult to predict (38).

Bronchoscopic drug delivery represents a promising alternative, offering several unique advantages and overcoming some limitations associated with the inhalation route. Although more invasive, therapeutic bronchoscopy provides direct access not only to peribronchial tumors, but also to deeper regions of the lung. This approach enables high local concentrations of chemotherapeutics, precise intratumoral injections at multiple sites within a lesion, and the co-administration of multiple agents. These characteristics make it particularly relevant for unresectable LC, where systemic therapy remains the only option. Moreover, bronchoscopy delivery bypasses common limitations of other local routes of administration, including particle deposition in the upper respiratory tract, irritation of upper airway tissues, and enzymatic degradation of biomolecules (28, 36, 37). Additionally, procedures such as EBUS-TBNI allow intratumoral delivery with high precision and low complication rates. Bronchoscopic delivery can also be integrated into diagnostic workflows combining visualization, sampling, and treatment, or with localized therapeutic interventions such as thermal ablation, cryotherapy, and brachytherapy, within a single session. Recent technological advances in interventional pulmonology, including electromagnetic navigation, robotic-assisted bronchoscopy, and ultrathin bronchoscopes, have further expanded the feasibility of reaching peripheral and deep-seated lesions. A comparative overview of the advantages and limitations of the different administration routes for lung treatment is provided in **Table 4**.

**Table 4:** Comparative overview of the advantages and limitations of different administration routes for lung treatment.

Route	Pros	Cons	Reference
Inhalation (DPI, MDI, Nebulizers)	Non-invasive administration Rapid delivery to the lungs Clinically convenient Reduced systemic exposure	User-dependent performance Heterogeneous aerosol deposition Limited applicability to biologics	(92, 93)
Bronchoscopy (Direct installation, BAL)	Site-specific drug delivery Allows sampling and real-time imaging Suitable for Theranostic applications	Requires trained personnel Invasive procedure	(94, 95)
Systemic (oral/IV)	Uniform dosage control Accessible monitoring Broad bioavailability	Lack of site specificity Potential systemic side effects Limited local concentration Surveillance requirement	(96)
Intratracheal instillation	Targeted deposition Regulated dosing in pre-clinical studies	Inconsistent absorption Technical variability Primarily adjunctive technique	(90)
Intranasal	Non-invasive administration Easily Administered treatment Expedited absorption Suitable for both local and systemic delivery	Limited dose volume Mucosal irritation Short residence time Formulation challenges	(91)

3. RNAs-therapies in Lung Cancer

RNA-based therapeutics are largely underexplored in lung cancer, although their application benefits from a strong pharmacological rationale. Their molecular mechanisms build on well-established pathways previously targeted by small-molecule inhibitors acting on the same oncogenic drivers. SiRNAs, in particular, have demonstrated therapeutic efficacy in several non-oncological indications, with an increasing number of clinical trials in recent years and six FDA-approved products to date. Innovations in biomaterials and nano- and micro-technologies have significantly accelerated the development of a number of RNA therapies, both in preclinical and in clinical investigations. Lipid nanoparticles (LNPs) (14), N-acetylgalactosamine (GalNAc) conjugates (15), microrobotic swarms (16), biodegradable polymers (17), SORT-NPs and stimuli-responsive NPs (18), have been shown to provide a safe, cost-efficient, and scalable platform for high-precision nucleic acid delivery (24).

These advancements in delivery system technologies, together with the potential of localized administration routes, further enhance the feasibility of developing and applying siRNA therapies in lung cancer. In this context, EBUS-TBNI could serve as a translational bridge, enabling the adaptation of intratumoral RNA delivery strategies to LC. Moreover, emerging technologies, including microneedle arrays fabricated from biodegradable materials and intralesional delivered siRNA–polymer conjugates, may improve local retention and expand the toolkit for precise pulmonary delivery (97, 98). Integrating these advances with bronchoscopic delivery could provide highly selective, non-systemic therapeutic options for LC patients.

As a concluding perspective, this review calls for a renewed vision that bridges RNA biology, nanobiotechnology, and interventional pulmonology to redefine therapeutic strategies in thoracic oncology. A close, active, and deeply integrated collaboration between scientists and clinicians, grounded in a shared awareness of its importance, is essential to advance medical progress.

## 6 Methods

*In-vivo* studies published between January 1, 2020, and July 30, 2025, were retrieved from PubMed, and Web of Science searching for the following keywords: “LC”, “siRNA-based therapies”, “Pulmonary delivery”, and “Orthotopic Models”.

Clinical studies published between January 1, 2020, and October 3, 2025, were retrieved from ClinicalTrials.gov, PubMed, and Web of Science. The search included both individual and combined terms related to local drug delivery. Specifically, the following keywords were used: “Inhalation”, “Bronchoscopy”, “EBUS-TBNI”, “siRNA”, “Lung Cancer”, “Chemotherapy”, and “Immunotherapy”.
